# Computational modeling of pro-inflammatory cytokine-enhanced blood coagulation

**DOI:** 10.64898/2026.05.02.722421

**Published:** 2026-05-04

**Authors:** Geli Li, Galit H. Frydman, He Li

**Affiliations:** 1 School of Chemical, Materials, and Biomedical Engineering, University of Georgia, Athens, GA 30602; 2 Division of Comparative Medicine, Department of Biological Engineering, Massachusetts Institute of Technology, Cambridge, MA, United States.; 3 Division of Trauma, Emergency Surgery, and Surgical Critical Care at the Massachusetts General Hospital, Boston, Massachusetts, United States.

**Keywords:** Computational modeling, pro-inflammatory cytokines, blood coagulation, COVID-19

## Abstract

The interplay between inflammation and coagulation is a central driver of thrombotic risk across various diseases. While mathematical models of blood coagulation are well established, there remains a critical gap in quantitative frameworks that capture inflammation-induced hypercoagulability. In this study, we develop a mathematical model that explicitly simulates the interaction between pro-inflammatory cytokines and the coagulation cascade. The model incorporates key mechanisms, including: (i) upregulation of tissue factor (TF) by IL-1*β*, IL-6, and TNF-*α*; (ii) suppression of natural anticoagulants, namely antithrombin III (ATIII) and tissue factor pathway inhibitor (TFPI), by IL-6 and TNF-*α*; and (iii) feedback amplification of proinflammatory cytokines by thrombin. By encoding the bidirectional feedback between inflammatory and coagulation pathways, the model captures essential features of inflammation-driven hypercoagulability and enables systematic quantification of how variability in inflammatory extent and duration results in heterogeneous thrombin generation (TG) dynamics. To evaluate its effectiveness, we integrate the model with TG assays and apply it to virtual patient cohorts representing four clinically distinct conditions: COVID-19, sickle cell disease (SCD), type 2 diabetes mellitus (T2DM) and Hemophilia A. Model simulations predict that disease-specific inflammatory environments induce distinct shifts in TG dynamics. In COVID-19 and T2DM, elevated cytokine levels lead to shortened lag times and increased thrombin peak, whereas in SCD, shortened lag times are accompanied by a reduced thrombin peak. These effects are strongly modulated by both cytokine concentration and duration of exposure. These results demonstrate that the proposed computational model augments conventional TG assays by mechanistically linking inflammatory signaling to disease-specific coagulation responses. Collectively, the proposed computational framework extends conventional TG assays by considering the interplay between inflammation and coagulation, thereby providing a potential tool for predicting disease progression and identifying disease-specific therapeutic targets to advance personalized management strategies in thrombo-inflammatory disorders.

## 1 Introduction

Human blood coagulation is a highly orchestrated biochemical cascade that protects the body from excessive bleeding following vascular injury [1]. Its central purpose is to limit blood loss by rapidly forming a clot at the site of damage. Hemostasis, the physiological process that governs this response, relies on a coordinated interplay among three major components: vascular regulation, activation of the coagulation cascade, and fibrinolytic dissolution of the clot. Proper balance among these mechanisms is essential for maintaining homeostasis. When this balance is disrupted, serious and potentially life-threatening conditions can arise. Insufficient levels or activity of coagulation factors, as seen in hemophilia A, or dysregulated fibrinolysis [2], can result in uncontrolled bleeding [3]. Conversely, excessive production of procoagulant or prothrombotic factors can drive inappropriate clot formation, leading to thrombosis [4]. Because both bleeding disorders and thrombotic events pose significant health risks, tight regulation of hemostasis is essential for maintaining physiological stability.

Inflammation is increasingly recognized as one of the key drivers of prothrombotic events across a wide range of pathological conditions [5, 6]. Inflammatory signaling perturbs hemostatic homeostasis by simultaneously upregulating procoagulant pathways and weakening endogenous anticoagulant and fibrinolytic safeguards [7, 8]. Mechanistically, pro-inflammatory cytokines and innate immune activation promote tissue factor (TF) expression in monocytes and endothelial cells, enhance platelet activation, and amplify thrombin generation (TG), thereby accelerating the initiation and propagation of clots [9–11]. In parallel, inflammation suppresses major anticoagulant pathways, including antithrombin III (ATIII), the protein C system, and the tissue factor pathway inhibitor (TFPI), and attenuates fibrinolysis (e.g., through impaired plasmin generation and increased fibrinolytic inhibition), further shifting the system toward a hypercoagulable state [12]. When excessive or sustained, this immunothrombosis could become maladaptive, driving microvascular thrombosis, end-organ hypoperfusion, and organ dysfunction in settings, such as sepsis, severe viral infection, and chronic inflammatory disease [13, 14].

Key inflammatory mediators, including tumor necrosis factor-*α* (TNF-*α*), interleukin-6 (IL-6), and interleukin-1*β* (IL-1*β*), can directly remodel the hemostatic phenotype of vascular and immune compartments. These cytokines induce TF expression in monocytes and endothelial cells and promote a procoagulant endothelial switch, providing a potent trigger for TF-dependent TG [15, 16]. Moreover, inflammation enhances endothelial dysfunction and platelet activation, and strengthens platelet–leukocyte/platelet–innate immune cell interactions, thereby amplifying thrombin formation through coupled cellular and plasma-protease mechanisms [17–20]. Such inflammation-driven coagulation abnormalities, often referred to as thromboinflammation/immunothrombosis, are widely observed in severe inflammatory diseases, including sepsis and COVID-19, where pathways linked to TF, platelets, and neutrophils contribute to systemic coagulopathy and thrombotic complications [21–23]. However, the dynamic, nonlinear, and feedback-rich nature of inflammation–coagulation crosstalk is not adequately captured by isolated or static biomarkers (e.g., single time-point cytokines, D-dimer, PT/aPTT). This limitation motivates quantitative and systems-level approaches, including mechanistic/QSP modeling of coagulation networks, to integrate multifactor interactions and characterize inflammation-induced prothrombotic states in a time-resolved manner [24].

Current mathematical models for simulating blood coagulation are categorized into two main types: ordinary differential equation (ODE) models and partial differential equation (PDE) models. The ODE models, designed to mimic thrombin and fibrin generation assays, operate on the principle that biochemical reactions can be represented as kinetic equations derived from experimental data [25–32]. These models focus on temporal changes, illustrating how concentrations of coagulation factors evolve over time under spatially uniform conditions. They are particularly useful for predicting concentration changes and identifying new reaction mechanisms, especially when there is a discrepancy between model predictions and actual data. In contrast, PDE-based models and other hybrid models are utilized to simulate thrombus growth, taking into account both unstirred systems and blood flow conditions [33–44]. These spatio-temporal models are essential for understanding clot growth within blood vessels, as they consider spatial concentration variations and can integrate blood flow velocity, often through a convection term, to provide deeper insights into the clot formation and growth dynamics [43, 44].

Despite significant advances in modeling coagulation pathways, existing mathematical frameworks remain insufficient to capture the complexity of inflammation-driven hypercoagulability. To address this gap, we propose a computational model that mechanistically links pro-inflammatory cytokines to coagulation factor dysregulation. Specifically, the model incorporates three clinically relevant interactions: 1) upregulation of TF expression by IL-1*β*, IL-6, and TNF-*α*; 2) cytokine-mediated suppression of the natural anticoagulants ATIII and TFPI by TNF-*α* and IL-6; and 3) thrombin-driven amplification of TNF-*α* and IL-6, reinforcing the thromboinflammatory feedback loop. We demonstrate the effectiveness of the proposed model by applying it to enhance TG assays across virtual patient cohorts representing four distinct disease states: COVID-19 coagulopathy, sickle cell disease (SCD), type 2 diabetes mellitus (T2DM), and hemophilia A. By rigorously quantifying how the pro-inflammatory cytokines alter coagulation dynamics across different hypercoagulable states, this work establishes a mechanistic framework for dissecting thromboinflammatory pathophysiology, with potential applications for predicting progression of inflammation-induced hypercoagulability and identifying disease-specific therapeutic targets to advance precision-medicine approaches to the management of thromboinflammatory disorders.

## 2 Data and Methods

### 2.1 Model structure

**Figure 1:**
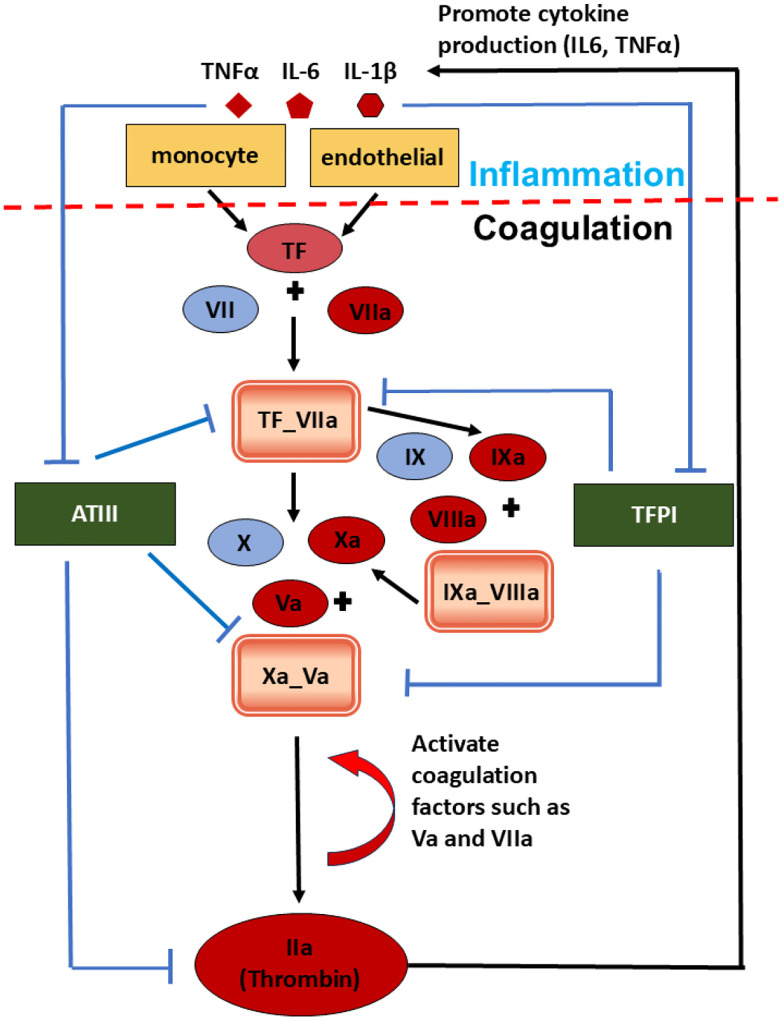
Schematic diagram of the model structure for simulating the interaction between inflammation cytokines and blood coagulation.

The model is organized into two functionally distinct but tightly coupled subsystems representing inflammation and coagulation. As illustrated in Fig. 1, the inflammation subsystem governs the time-dependent variation of IL-6, IL1-*β*, and TNF-*α*, while the coagulation subsystem captures TF-initiated TG. Within the model, inflammatory activity acts upstream of coagulation by promoting TF availability and attenuating endogenous anticoagulant regulation. Specifically, IL-6, IL1-*β*, and TNF-*α* activated monocytes and endothelial cells, thereby enhancing TF expression that triggers initiation of the coagulation cascade. On the other hand, pro-inflammatory cytokines also could suppress the natural anticoagulants, including ATIII and TFPI, shifting the hemostatic balance toward a prothrombotic state. The coagulation subsystem generates thrombin as a central output, which could subsequently activate platelets and promote the generation of a cross-linked fibrin network, two key components of thrombosis. In addition, thrombin feeds back into the inflammatory subsystem by promoting the production of IL-6, IL1-*β*, and TNF-*α*. This feedback establishes a positive amplification loop in which inflammation enhances TG while thrombin, in turn, reinforces inflammatory signaling. Such bidirectional coupling provides a mechanistic framework for simulating sustained thromboinflammatory activation.

The coagulation module is built upon the mechanistic framework of Hockin et al. [29], which describes the core TF-initiated coagulation cascade and remains one of the most widely validated ODE-based models of hemostasis. The inflammation module incorporates three key proinflammatory cytokines, namely, IL-6, IL-1*β*, and TNF-*α*, whose interactions with coagulation factors form the mechanistic basis of thromboinflammatory coupling. The molecular pathways linking inflammatory mediators to TF upregulation, primarily through monocyte and endothelial cell activation [45, 46], and those linking thrombin to cytokine amplification via PAR-1/NF-*κ*B signaling [47, 48] are inherently intracellular, involving complex transcriptional and post-translational regulation that cannot be resolved at the level of a systems-scale ODE model. We therefore adopt a phenomenological modeling strategy, constructing quantitative input-output relationships between: 1) cytokine concentrations (IL-1*β*, IL-6, TNF-*α*) and TF production, and 2) thrombin concentration and TNF-*α*/IL-6 amplification. These relationships are parameterized directly from published experimental data, ensuring biological feasibility without requiring explicit representation of intracellular signaling intermediates.

To determine the production and degradation rates of each inflammatory species, we leveraged experimentally reported half-life values: IL-1*β* and IL-6 each carry a plasma half-life of approximately 1 hour [49, 50], while TNF-*α* is cleared considerably faster, with a half-life of only 4.6 minutes [51]. First-order metabolic degradation rates were derived directly from these half-lives. Production rates were then computed by imposing a steady-state constraint: in the absence of disease or external perturbation, each species must be maintained at its reported normal plasma concentration. This homeostatic calibration ensures that the model is physiologically grounded at baseline and that any simulated perturbations reflect disease-specific deviations from normal hemostatic and inflammatory tone, rather than artifacts of parameter initialization.

Overall, the proposed mathematical model simulates 38 reactions involving a total of 44 species and 72 parameters. Details of the model reaction and parameters are included in the supplementary materials. All model data, including reaction rules, species, reaction rates, and parameter values, were processed using MATLAB’s Simbiology Toolbox (MathWorks, Natick, MA). Since our model simulates a variety of cytokine and coagulation stimulation conditions, we converted all dosing units used in *in vitro* experiments (often in ng/ml) into mol per liter. ImageJ software (NIH) [52] and Getdata were utilized in the analysis and quantitative digitization of Western blot and other experimental datasets.

### 2.2 Clinical data for creating virtual patients

We generated virtual patient cohorts to investigate how the extent and duration of inflammation alter TG dynamics across four distinct diseases: COVID-19–associated coagulopathy, SCD, T2DM, and hemophilia A. Clinical laboratory ranges for coagulation factors and inflammatory markers were collected for each disease group, as summarized in Table 1. To construct these cohorts, we employed Latin hypercube sampling to efficiently explore parameter spaces and generate physiologically diverse virtual patients. Using disease-specific distributions (mean and standard deviation) of coagulation factors and cytokines as reference values, we generated 2,000 virtual patients per condition. We further calibrated the sampled parameter sets using key TG metrics, namely lag time, time to peak, and peak thrombin, derived from experimental TG data for each disease group [53–56]. This calibration step ensured that the simulated patients exhibited physiologically consistent coagulation profiles aligned with observed TG dynamics. Finally, using the TG curve as a quantitative biomarker, we systematically analyzed how pro-inflammatory cytokines modulate TG dynamics across the four disease states, thereby elucidating disease-specific thrombo-inflammatory mechanisms.

## 3 Results

### 3.1 Model calibration

**Figure 2:**
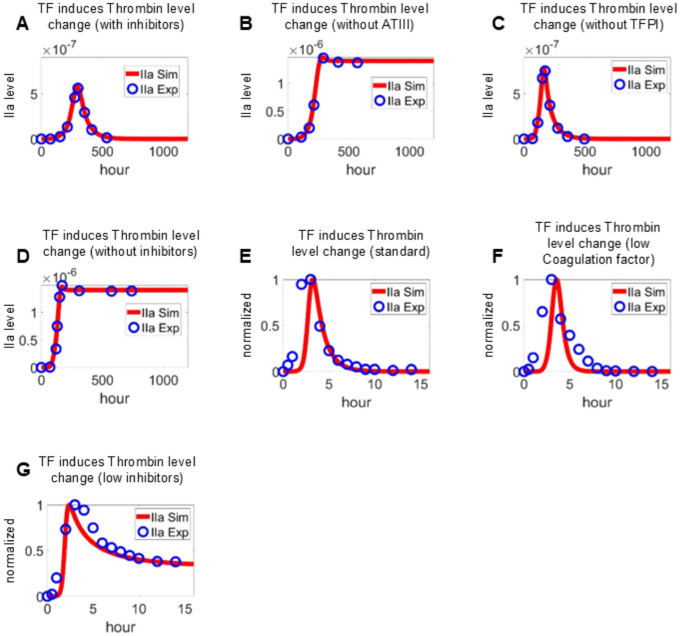
Calibration of coagulation module using TF–driven TG curves under various inhibitory conditions. TG induced by TF in the presence of ATIII and TFPI (A), in the absence of ATIII (B), in the absence of TFPI (C), in the absence of both ATIII and TFPI (D). Experimental data used in (A-D) is adopted from [29]. TG initiated by factor VIIa-TF in the presence of all proteins at mean plasma concentrations (E), in the presence of procoagulants (prothrombin, factors V, VIII, IX, and X) at 150% and anticoagulants (AT-III and TFPI) at 50% of their mean plasma concentrations (F), and in the presence of procoagulants at 50% and anticoagulants at 150% of their mean plasma concentrations (G). Experimental data used in (E-G) is adopted from [69].

First, we calibrate the coagulation module independently against both reference model simulations and experimental TG data under multiple perturbation conditions as reported in [29]. Figs. 2(A–C) compare thrombin dynamics simulated by the coagulation module (red curves) with those generated by a reference coagulation model under the same 25pM TF stimulations (blue circles). Specifically, thrombin responses were evaluated in the presence of ATIII and TFPI (Fig. 2(A)), in the absence of ATIII (Fig. 2(B)), in the absence of TFPI (Fig. 2(C)), in the absence of both ATIII and TFPI (Fig. 2(D)), respectively. Across all conditions, the coagulation module closely reproduced the results of the reference model, capturing key dynamical features including thrombin initiation timing, peak amplitude, and sustained or decaying response profiles. In particular, perturbations to anticoagulant pathways produced consistent qualitative and quantitative effects, indicating that the coagulation module preserves the essential regulatory structure of the reference framework [29].

To evaluate the robustness and physiological fidelity of the model, we also simulated TG dynamics under conditions corresponding to separate experimental measurements in normal plasma reported by [69]. Model predictions were compared against three experimental scenarios reflecting systematic perturbations in procoagulant and anticoagulant factor levels. Thrombin generation was initiated with 5 pmol/L factor VIIa–TF in the presence of all coagulation proteins under the following conditions: (1) baseline, with all factors at mean plasma concentrations; (2) a procoagulant-dominant state, with elevated levels (150%) of prothrombin and factors V, VIII, IX, and X, combined with reduced levels (50%) of natural anticoagulants, including ATIII and TFPI; and (3) an anticoagulant-dominant state, with reduced procoagulant levels (50%) and elevated anticoagulant levels (150%). As shown in Fig. 2(E), the model accurately reproduced the characteristic thrombin burst observed under physiological conditions, followed by a rapid decay phase. In the anticoagulant-dominant setting, both simulations and experimental data demonstrated delayed onset and attenuated peak thrombin generation (Fig. 2(F)), consistent with impaired propagation of the coagulation cascade. Conversely, in the procoagulant-dominant condition, reduced inhibitory capacity led to sustained thrombin activity with prolonged decay kinetics (Fig. 2(G)). Overall, the close agreement between simulated and experimental TG profiles across these perturbations supports the model’s ability to capture the dynamic balance between procoagulant drivers and endogenous inhibitors.

**Figure 3:**
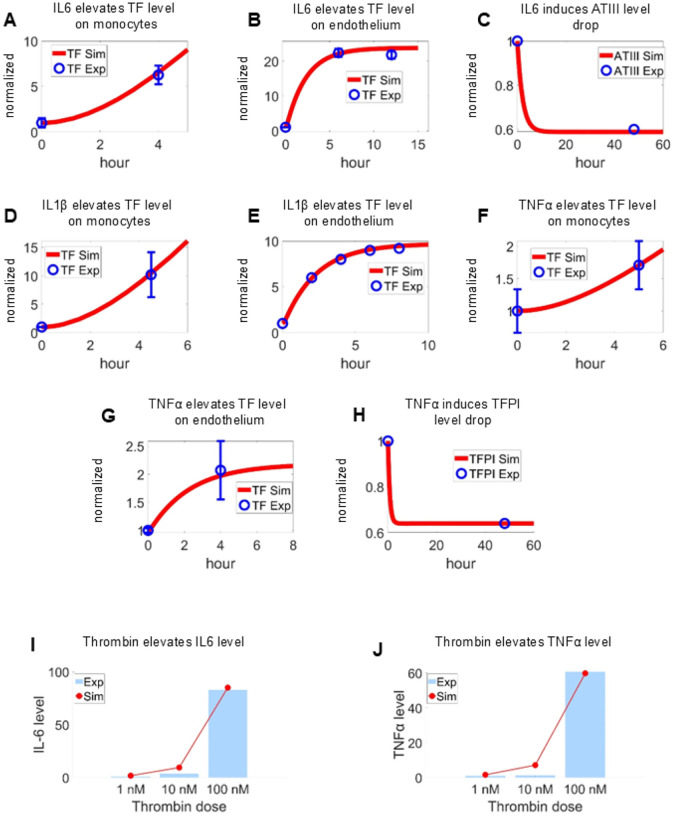
Calibration of inflammation–coagulation coupling mechanisms. (A–C) IL-6–induced TF upregulation and ATIII suppression over time [70, 71, 76]. (D–E) IL-1*β*–induced TF expression dynamics [72, 73]. (F–H) TNF-*α*–induced TF upregulation and TFPI suppression [74, 75, 77]. (I–J) Thrombin-induced cytokine production, showing dose-dependent induction of IL-6 and TNF-*α* [78]. Model simulations (red lines) were calibrated against experimental measurements (blue markers), with all outputs normalized to baseline levels.

Next, we calibrated the interplay between inflammation and coagulation modules using extensive experimental datasets to constrain parameters governing the interactions between cytokines and the coagulation factors. Specifically, cell-type–specific data were used to calibrate the how the IL-6 (Figs. 3(A and B)) [70, 71]), IL-1*β* (Figs. 3(D and E)) [72, 73] and TNF-*α* (Figs. 3(F and G)) [74, 75] enhance the expression of TF on monocytes and endothelium. Furthermore, motivated by experimental observations that excessive inflammatory signaling can suppress endogenous anticoagulant pathways [76, 77], we incorporated cytokine-mediated inhibition of ATIII and TFPI into the calibration process, as shown in Figs. 3(C and H). This negative regulatory mechanism was included to capture the procoagulant shift observed under high inflammatory burden. Moreover, the activating effect of thrombin on cytokine production was calibrated using experimental data obtained at different thrombin concentrations as shown in Figs. 3 (I–J) [78], enabling the model to represent feedback amplification from coagulation and inflammatory signaling.

### 3.2 Quantify the impact of pro-inflammatory cytokines on coagulation

To investigate the effects of varying inflammation intensities on coagulation, we employed two complementary biomarkers: the thrombin–antithrombin complex (ATIII-IIa) and the TG curve. The TG curve was used to characterize the kinetics of TG under cytokine-mediated perturbation, while generation of ATIII-IIa reflects the net amount of thrombin generation. Inflammatory stimulation was modeled by varying the concentrations of key pro-inflammatory cytokines at 0.5, 1, 2, 5, 10, and 50 times of their baseline values, spanning the range of cytokine levels documented in the clinical literature (Table 1). These variations represent a physiologically and clinically relevant spectrum of inflammatory states, from sub-physiologic to severely hyperinflammatory conditions. Notably, since the majority of COVID-19 cohort studies report a maximum IL-1*β* elevation of approximately 10-fold, IL-1*β* concentrations were capped at a 10× increase in all simulations to maintain clinical relevance.

Because coagulation and inflammation operate on distinct timescales: coagulation cascades unfold within seconds to minutes, whereas cytokine-mediated modulation of coagulation factor expression and endothelial activation evolves over hours, we modeled inflammatory impact on coagulation factors over 12-hour and 24-hour periods prior to initiating the coagulation module. This approach allowed sufficient time for inflammatory mediators to activate monocytes and endothelial cells, upregulating TF expression and thereby modulating the extent of thrombin generation.

**Figure 4:**
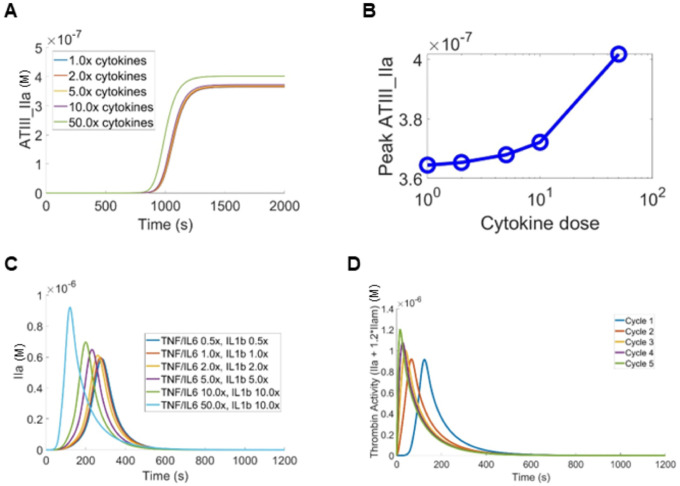
Simulated effects of pro-inflammatory cytokines on coagulation dynamics. (A)The dynamics of ATIII-IIa generation by different levels of cytokines. (B) The total amount of ATIII-IIa generated by different levels of cytokines. (C) The TG dynamics at different levels of cytokines. In (A-C), cytokine concentrations were scaled relative to the normal baseline condition. (D) Sustained inflammatory exposure at the baseline condition further enhanced thrombin activity, indicating time-dependent reinforcement of inflammation–coagulation coupling.

In the simulations, we initiated thrombin generation with 5 pmol/L TF, consistent with established experimental conditions, to define a baseline coagulation response. Under this setting, TG was computed in the absence of pro-inflammatory cytokine modulation, providing a baseline against which the effects of proinflammatory cytokines on coagulation dynamics could be systematically evaluated. Fig. 4(A) displays the time-course of ATIII-IIa complex accumulation across cytokine doses (1×–50×), where all conditions follow a sigmoidal accumulation pattern, with a sharp transition occurring between 750–1,000 seconds. Higher cytokine concentrations produce a modest but consistent upward shift in plateau ATIII-IIa levels, suggesting that inflammation augments total thrombin generation without dramatically altering the kinetic signature. It is noted that the 1× and 2× curves are nearly superimposable, implying that a minor to mild inflammation does not meaningfully perturb net thrombin production. Quantification of the peak ATIII-IIa as a function of cytokine dose in Fig. 4(B) shows that the peak ATIII-IIa rises non-linearly, with the most dramatic increase occurring between 10× and 50×, suggesting a procoagulant tipping point at severe hyperinflammation. The relatively flat response between 1×–10× implies that mild to moderate inflammatory states, such as at the early stage of COVID-19 or sepsis, may not substantially elevate ATIII-IIa alone, potentially limiting its sensitivity as an early biomarker at lower inflammation intensities.

Fig. 4(C) illustrates the temporal dynamics of thrombin generation across the full range of cytokine doses, revealing a clear dose-dependent response. As cytokine concentrations increase from 0.5× to 10×, peak thrombin levels rise progressively. At higher inflammatory stimulation (TNF-*α* and IL-6) at 50×, thrombin increases sharply, highlighting the amplifying effect of severe inflammation on coagulation potential. Meanwhile, the time-to-peak shortens with increasing cytokine dose, indicating accelerated initiation and propagation of coagulation. This behavior is mechanistically consistent with cytokine-driven upregulation of TF expression on activated monocytes and endothelial cells, a key pathway linking inflammation to enhanced thrombin generation.

### 3.3 Simulate the amplifying cycles of inflammation and coagulation

Thrombin generation from coagulation exerts a positive feedback effect on inflammatory mediator production via PAR-1/NF-*κ*B signaling [47, 48], perpetuating the inflammation–thrombosis cycle. To capture this cyclical behavior, simulations were structured as iterative two-phase sequences: the inflammation module was executed for either 12 hours, representing sustained inflammatory states respectively, followed by a 1,200-second coagulation module. Upon completion of each coagulation phase, the inflammation module was reinitiated using the thrombin exposure metric derived from that cycle, allowing inflammatory mediator concentrations to evolve in response to the coagulation output. Coagulation factor concentrations were restored to their initial values between cycles to establish a defined computational baseline. This two-phase framework enabled systematic analysis of how inflammation-driven hypercoagulation manifests across patient cohorts with differing extent and duration of inflammation.

Fig. 4(D) shows thrombin activity across five continuous inflammation–coagulation cycles under sustained inflammatory conditions, and the results show that thrombin activity is progressively reinforced over successive cycles, indicating a cumulative amplification effect driven by prolonged inflammatory stimulation. This pattern suggests that sustained or unresolved inflammation, as opposed to a single acute inflammatory insult, is the critical determinant of escalating coagulation activity, consistent with the clinical trajectory observed in severe COVID-19 and sepsis, where persistent cytokine elevation correlates with disease worsening [9, 79, 80]. The multi-cycle amplification supports the concept that each inflammation-coagulation cycle drives the system to a more procoagulant response in the subsequent cycle, through upregulation of TF on monocytes and endothelial cells, sustained elaboration of pro-inflammatory cytokines, and suppression of endogenous anticoagulant pathways. Clinically, these results could mimic the self-reinforcing thromboinflammatory loop that underlies microvascular thrombosis and organ dysfunction in hyperinflammatory states, underscoring why early interruption of the inflammatory stimulus, rather than anticoagulation alone, may be essential to breaking the cycle [19, 81].

### 3.4 Enhance TG assays across various virtual patient cohorts

In this section, we employ the proposed model to enhance TG assays across virtual patient cohorts representing four distinct disease states: COVID-19 coagulopathy, SCD, T2DM, and hemophilia A. We first apply the coagulation factor ranges listed in Table 1 to the coagulation module and compare four commonly used metrics derived from the generated TG curves, namely lag time, time-to-peak (TTP), peak thrombin concentration, and endogenous thrombin potential (ETP) against experimentally reported values for COVID-19 coagulopathy [53], SCD [55], T2DM [54], and hemophilia A [56]. We observe that the model predictions do not agree with the TG dynamics for any of the four examined disease conditions. These findings echoed with previous studies assessing the performance of existing *in silico* coagulation models for predicting TG in normal subjects, which indicate that calibration of the coagulation module is necessary before it can reliably reproduce the key TG metrics.

**Figure 5:**
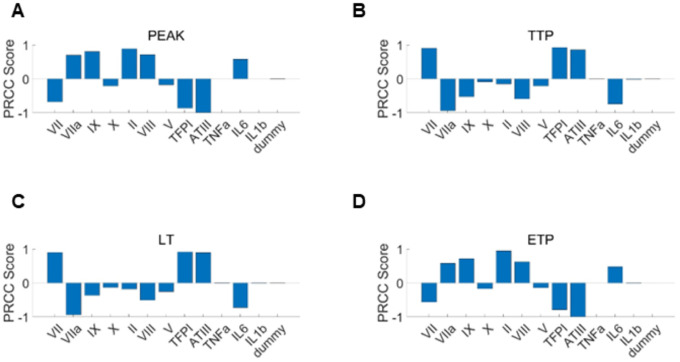
Sensitivity analysis of inflammatory and coagulation factors on the TG dynamics. Global sensitivity analysis using partial rank correlation coefficients quantified the influence of coagulation factors, anticoagulants, and inflammatory mediators on TG metrics, including Peak (A) time to peak (TTP, B), lag time (LT, C), and endogenous thrombin potential (ETP, D). The dummy variable acts as a control, representing a parameter with zero influence on the model output.

To calibrate the model effectively and efficiently, we first perform a sensitivity analysis using the Latin Hypercube Sampling (LHS) method to compute Partial Rank Correlation Coefficient (PRCC) values across the coagulation factor variation ranges from the four disease groups as listed in Table 1, following the algorithm described by Marino et al. [82]. The analysis targets four TG metrics: lag time, TTP, peak thrombin concentration, and ETP. For each disease group, 5,000 simulations were performed. The relative influence of each coagulation and inflammatory factor on these metrics for COVID-19 virtual patients was summarized in Figs. 5(A–D). Our results show that factor II concentration exerted the strongest positive effect on peak thrombin levels (Fig. 5(A)) and ETP (Fig. 5(D)), whereas ATIII showed the strongest negative association with both endpoints. Inflammatory mediators also displayed positive effects on peak and ETP, with IL-6 exhibiting a more pronounced influence than the other two inflammatory factors considered. Notably, although Factor VII is expected to positively regulate TG, PRCC analysis revealed a strong negative association with peak thrombin and ETP. This contradiction may arise from the structure of the Hockin model [29], in which excessive Factor VII leads to increased sequestration by TF, thereby limiting formation of the active VIIa–TF complex. The resulting reduction in effective VIIa–TF availability attenuates downstream coagulation cascade activation, ultimately diminishing thrombin peak and ETP. Consistent trends were observed for temporal TG metrics. Procoagulant and inflammatory factors predominantly exerted negative effects on TTP (Fig. 5(B)) and lag time (Fig. 5(C)), corresponding to accelerated TG, with Factor VIIa demonstrating the strongest time-shortening influence.

**Figure 6:**
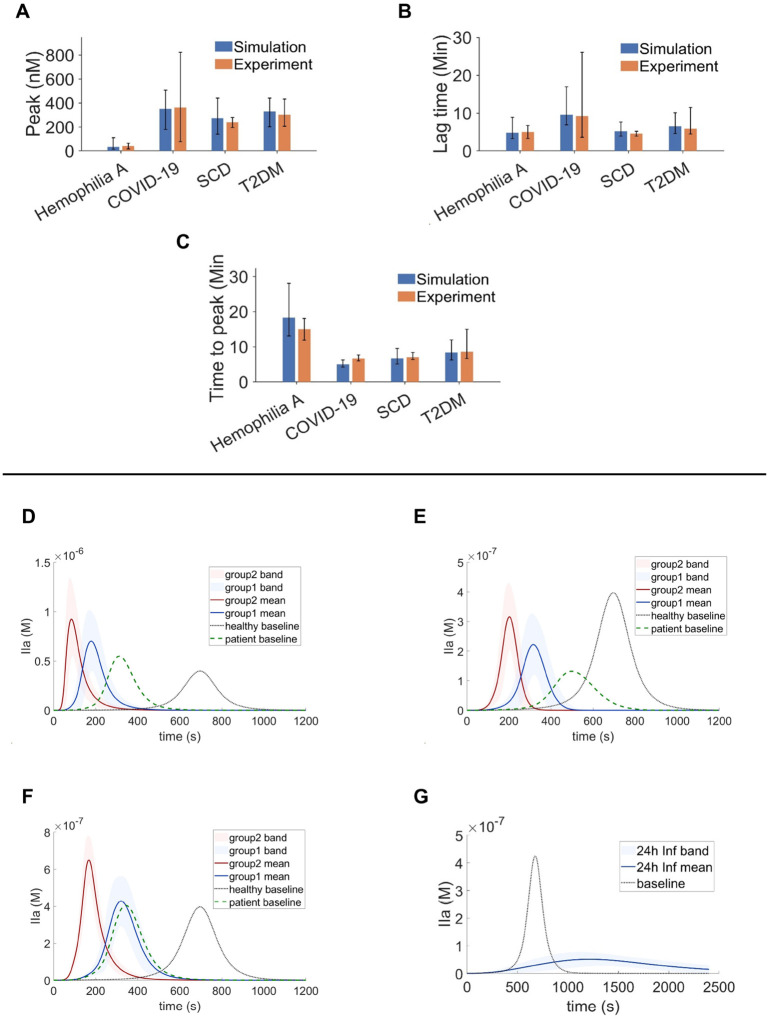
TG dynamics of virtual patients under four disease conditions. Comparison of simulated and experimental TG metrics, including peak (A), lag time (B), and time to peak (C), across hemophilia A, COVID-19, SCD, and T2DM. 2000 virtual patients are generated for each disease condition. TG profiles of virtual patients of COVID-19 (D), SCD (E), T2DM (F) under baseline conditions and inflammatory exposure (12 h and 24 h), shown as mean trajectories with variability bands. (G) TG profiles of virtual patients of Hemophilia A under baseline conditions. Virtual patient ensembles were constructed by sampling coagulation and inflammatory parameters within disease-specific physiological ranges.

Based on these sensitivity analysis results, we narrowed the variation ranges of the three most influential coagulation factors when generating virtual patients for each disease such that the key TG metrics predicted align with the experimentally measured values reported in [53–56], while remaining within physiologically and clinically relevant bounds. Figs. 6(A–C) compare the simulated TG metrics against corresponding experimental measurements across the four examined patient groups, and the results show that simulated TG metrics generally fell within the experimental variability ranges for each disease group, demonstrating that the proposed virtual patient generation pipeline can quantitatively capture the observed TG dynamics across distinct disease states. These results will be used as baseline conditions for downstream analysis of the impact of pro-inflammatory cytokines.

Next, we examine the impact of pro-inflammatory cytokines on TG curves for virtual patient groups representing COVID-19 (Fig. 6(D)), SCD (Fig. 6(E)), and T2DM (Fig. 6(F)). Proinflammatory cytokine exposure was simulated at two durations, namely 12 hours and 24 hours, to assess how exposure length modulates coagulation response. For COVID-19 (Fig. 6(D)), both 12h and 24h inflammation exposures produce a dramatically elevated thrombin peak relative to both the uninflamed baseline and normal subjects, accompanied by shortened lag time and earlier time-to-peak. This pattern is consistent with the well-documented hypercoagulable state in COVID-19, driven by cytokine-mediated activation of coagulation pathways. Notably, the 24h exposure amplifies thrombin generation considerably beyond the 12h case, indicating a durationdependent inflammatory response. In SCD (Fig. 6(E)), inflammation similarly shortens lag time and advances peak formation; however, the peak thrombin magnitude remains lower than that observed in normal subjects. This apparent paradox likely results from the chronically depleted plasma Factor X pool characteristic of SCD. Ongoing low-extent coagulation activity in SCD may consume Factor X at a rate that exceeds hepatic replenishment [83, 84], thereby constraining the maximum thrombin burst even under inflammatory stimulation.

For T2DM (Fig. 6(F)), the TG curves qualitatively mirror those of COVID-19, exhibiting shortened lag time, earlier peak formation, and elevated TG peak relative to the uninflamed baseline and normal subjects, collectively indicating inflammation-driven enhancement of coagulation activity. Across all three conditions, the magnitude of these effects is mounted with exposure duration: prolonged inflammatory stimulation (24h) consistently produces earlier thrombin onset and higher peak IIa levels than shorter exposure (12h). In contrast to the above three diseases, the hemophilia A virtual patient population (Fig. 6(G)) exhibited a distinct response pattern. Under baseline conditions (without inflammatory signaling), TG remained markedly attenuated, consistent with a hypo-coagulable phenotype. This constrained thrombin amplification reflects a dominant effect imposed by coagulation factor VIII deficiency. Together, these results demonstrate that TG-calibrated virtual patient populations capture both inflammation-driven hypercoagulability mechanisms and disease-specific constraints on TG.

## 4 Discussion and Summary

In this study, we developed and calibrated a mechanistic inflammation-coagulation model and employed it in a virtual patient framework to investigate how inflammatory burden reshapes coagulation under hyperinflammatory conditions [9, 10, 85]. The model encodes cytokine-mediated upregulation of TF in monocytes and endothelial cells, concurrent suppression of endogenous anticoagulants, specifically ATIII and TFPI, and positive feedback from thrombin to inflammatory signaling [15, 86–89], which enables quantification of how the increasing inflammatory intensity and duration systematically alters TG at different disease conditions [15, 23, 90, 91]. Our results show that the virtual patient simulations reproduce trends qualitatively agree with clinical observations across different hypercoagulable diseases, including COVID-19, SCD and T2DM, demonstrating the feasibility of the model in simulating thrombo-inflammatory scenarios [23, 90–92]. Furthermore, our model shows that the interplay between inflammation and coagulation could promote a self-amplifying loop that leads to sustained thrombo-inflammatory conditions [85, 88]. This systems-level perspective provides a mechanistic explanation for why inflammatory states are frequently accompanied by heightened coagulation activity and thrombotic risk in severe inflammatory contexts [93, 94].

The inflammation intensity analysis in Figs. 4(A–C) highlights the nonlinear nature of inflammation–coagulation coupling. The increasing inflammatory burden leads to an exponential increase in TG, suggesting that coagulation activation is disproportionately sensitive to high cytokine levels. Importantly, virtual patient simulations demonstrate that both inflammatory intensity and duration critically impact the coagulation dynamics. Prolonged inflammatory stimulation resulted in earlier thrombin initiation and higher peak levels in COVID and T2DM patients compared with those with shorter exposure. These findings emphasize the importance of cumulative inflammatory burden rather than transient cytokine elevations, aligning with clinical observations linking sustained inflammation to progressive hypercoagulability [95, 96].

Disease-specific simulations revealed both shared and divergent TG dynamics under inflammatory conditions. In inflammatory diseases such as COVID-19 and T2DM, inflammatory signaling markedly amplified TG and accelerated coagulation initiation, consistent with clinical and experimental evidence of hypercoagulability in these settings [93, 97–99]. In contrast, the hemophilia A virtual population exhibited fundamentally attenuated TG dynamics, driven by factor VIII deficiency that constrains thrombin generation irrespective of inflammatory stimulus. This divergent behavior underscores the model’s capacity to capture a key mechanistic distinction between diseases, in which inflammation acts as the primary driver of coagulation dysregulation, and those in which an intrinsic factor deficiency suppresses the system-level coagulation activities.

Several limitations should be acknowledged. First, the model focuses on TG as a surrogate marker of coagulation activation without extending to the downstream consequences of thrombin activity, such as fibrin clot formation and propagation. Consequently, it cannot capture the full progression from coagulation initiation to thrombus formation and subsequent fibrinolysis. Second, the inflammatory module is built around a limited subset of cytokines, yet the *in vivo* inflammatory milieu is considerably more complex. Additional mediators, such as IL-12 and interferon-gamma (IFN-*γ*), have been reported to contribute to hypercoagulable conditions [100–102]. Due to this limitation, the model may underestimate or misrepresent inflammation–coagulation coupling in settings where these non-modeled mediators play a dominant role. Third, the TG data used for calibration of virtual patients were obtained from different laboratories employing distinct experimental protocols, which may introduce inter-laboratory variability that limits the feasibility of direct cross-disease comparison of thrombin generation dynamics.

In summary, this study introduces a mechanistic, TG-calibrated virtual patient framework that elucidates how inflammatory burden shapes coagulation dynamics. By incorporating interindividual variability, the proposed computational approach extends conventional TG assays to account for the interplay between inflammation and coagulation. This framework offers a potential tool for predicting disease progression, identifying disease-specific therapeutic targets, and supporting personalized management strategies in thrombo-inflammatory disorders.

## Figures and Tables

**Table 1:** Range of coagulation and cytokine factors for Normal subjects and subjects under four diseased conditions.

	COVID-19	T2DM	SCD
IL-6	232(1 − 32768)pg/ml [57]	4.1 (2.3 − 5.6) pg/mL [58]	60± 7pg/ml [59]
TNF*α*	25(1 − 1000)pg/ml [57]	3.8 (3.0 − 5.0) pg/mL [58]	122.3± 16.3pg/ml [60]
IL1 *β*	0.8(0 − 32)pg/ml [57]	0.2 (0.2 − 0.3) pg/mL [58]	3.5 (0.0 − 27.26) pg/mL [61]
VII	0.5mg/L (55%–170%) [62]	0.5mg/L (60% − 130%) [63]	0.31mg/L(±7.5%) [64]
VIIa	0.2mg/L (60% − 130%) [63]	0.2165mg/L±26.72% [65]	0.2mg/L (60% − 130%) [63]
IX	7.5mg/L ±50.6% [62]	5mg/L (60% − 130%) [63]	5mg/L (60% − 130%) [63]
X	11.1mg/L ±27.9% [62]	10mg/L (60% − 130%) [63]	6.6mg/L ±10% [64]
II	107.5 mg/L ±31.2% [62]	100mg/L (60% − 130%) [63]	75 mg/L ±10% [64]
VIII	0.262 mg/L±102.9% [62]	0.1mg/L (50% − 150%) [63]	0.1mg/L (50% − 150%) [63]
V	11.7mg/L ±44.9% [62]	10mg/L (60% − 130%) [63]	7.25mg/L ±17.5% [64]
TFPI	70ng/mL [66]	197.56 ± 94.88(pg/ml) [65]	70ng/mL [66]
ATIII	0.134mg/L ±15.1% [62]	0.15mg/L [67]	0.15mg/L [67]
	Hemopilia	Normal	
IL-6	-	-	
TNF*α*	-	-	
IL1 *β*	-	-	
VII	0.5mg/L (60% − 130%) [63]	0.5mg/L (60% − 130%) [63]	
VIIa	0.2mg/L (60% − 130%) [63]	0.2mg/L (60% − 130%) [63]	
IX	5mg/L (60% − 130%) [63]	5mg/L (60% − 130%) [63]	
X	10mg/L (60% − 130%) [63]	10mg/L (60% − 130%) [63]	
II	100mg/L (60% − 130%) [63]	100mg/L (60% − 130%) [63]	
VIII	< 1% [68]	0.1mg/L (50% − 150%) [63]	
V	10mg/L (60% − 130%) [63]	10mg/L (60% − 130%) [63]	
TFPI	70ng/mL [66]	70ng/mL [66]	
ATIII	0.15mg/L [67]	0.15mg/L [67]	
